# Development of a maxillofacial virtual surgical system based on biomechanical parameters of facial soft tissue

**DOI:** 10.1007/s11548-022-02657-5

**Published:** 2022-05-15

**Authors:** Mengjia Cheng, Yu Zhuang, Hanjiang Zhao, Meng Li, Lingfeng Fan, Hongbo Yu

**Affiliations:** 1 Department of Oral and Cranio-Maxillofacial Surgery, Shanghai Ninth People’s Hospital, College of Stomatology, Shanghai Jiao Tong University School of Medicine, Shanghai, 200011 China; 2 National Clinical Research Center for Oral Diseases, Shanghai, 200011 China; 3 Shanghai Key Laboratory of Stomatology & Shanghai Research Institute of Stomatology, Shanghai, 200011 China; 4 Department of Radiology, Shanghai Ninth People’s Hospital, College of Stomatology, Shanghai Jiao Tong University School of Medicine, Shanghai, 200011 China

**Keywords:** Maxillofacial soft tissue, Virtual surgery, Physical model, Insertion and cutting, Biomechanical properties, Haptic feedback

## Abstract

**Purpose:**

Lack of biomechanical force model of soft tissue hinders the development of virtual surgical simulation in maxillofacial surgery. In this study, a physical model of facial soft tissue based on real biomechanical parameters was constructed, and a haptics-enabled virtual surgical system was developed to simulate incision-making process on facial soft tissue and to help maxillofacial surgery training.

**Methods:**

CT data of a 25-year-old female patient were imported into Mimics software to reconstruct 3D models of maxillofacial soft and skeletal tissues. 3dMD stereo-photo of the patient was fused on facial surface to include texture information. Insertion and cutting parameters of facial soft tissue measured on fresh cadavers were integrated, and a maxillofacial biomechanical force model was established. Rapid deformation and force feedback were realized through localized deformation algorithm and axis aligned bounding box (AABB)-based collision detection. The virtual model was validated quantitatively and qualitatively.

**Results:**

A patient-specific physical model composed of skeletal and facial soft tissue was constructed and embedded in the virtual surgical system. Insertion and cutting in different regions of facial soft tissue were simulated using omega 6, and real-time feedback force was recorded. The feedback force was consistent with acquired force data of experiments conducted on tissue specimen. Real-time graphic and haptic feedback were realized. The mean score of the system performance was 3.71 given by surgeons in evaluation questionnaires.

**Conclusion:**

The maxillofacial physical model enabled operators to simulate insertion and cutting on facial soft tissue with realization of realistic deformation and haptic feedback. The combination of localized deformation algorithm and AABB-based collision detection improved computational efficiency. The proposed virtual surgical system demonstrated excellent performance in simulation and training of incision-making process.

**Supplementary Information:**

The online version contains supplementary material available at 10.1007/s11548-022-02657-5.

## Introduction

Performing incisions on facial soft tissue is one of the most common but delicate procedures for maxillofacial surgeons. Incisions are required to be performed layer by layer in order to avoid accident injury of nerves or blood vessels, so surgeons must control the depth of incisions precisely. Proper and smooth incisions are critical for flap acquisition and scar reduction in maxillofacial surgery [1]. Therefore, making incisions on facial soft tissue requires high level of skill and dexterity.

However, in traditional training, novice surgeons obtain surgical skills through practicing on synthetic materials, animals, cadavers, progressively operating on patients supervised by experienced surgeons. Mastering surgical skills takes a long training period because of scarce resources and limited opportunities [2, 3]. Virtual reality (VR) technology provides us a more flexible, efficient and lower-cost manner for surgical training. In virtual surgical simulation, three-dimensional (3D) virtual anatomic models combined with biomechanical properties are constructed to simulate patients; Deformation and collision detection algorithms are designed to realize interaction between virtual instruments and anatomic structures; haptic device can give trainees real tactile feedback [4]. Therefore, VR-based simulation system can provide operators secure, reusable virtual models and immersive, interactive practice environment.

In recent years, a series of virtual surgical systems have been developed to simulate maxillofacial surgery. Most of them targeted to bone operations such as drilling on the jaws [5–7], sawing or osteotomy in orthognathic surgery [6–8], moving bone segments [9–11], mandibular angle reduction [12, 13], dental implant surgery (stepwise drilling) [14], bone fractures reduction [15] and bone defects reconstruction [16]. Few virtual surgical systems were incorporated with facial soft tissue models or were developed specially for soft tissue surgery [17, 18]. Simulation of facial soft tissue surgery was hindered for the reason that facial soft tissue exhibits complex properties of nonlinearity, anisotropy, non-uniform viscoelasticity. Real deformation and experimental force data are insufficient. Therefore, in our previous work [19], a series of experiments on fresh human cadavers were conducted to investigate characteristics of facial soft tissue. Cutting forces were acquired and insertion curves were fitted as polynomial equations.

This study aimed to construct a maxillofacial biomechanical force model based on previous experimental data and develop a haptics-enabled virtual surgical system to simulate incision-making process on facial soft tissue and realize real-time deformation and haptic feedback.

### Related works

Lots of strategies of soft tissue modeling for cutting simulation were proposed. Soft tissue was originally modeled with meshes, and incisions were rendered by element removal or splitting or subdivision [20]. However, mesh refresh causes generation of deformed meshes and system instability. Meshless methods in which soft tissue is modeled with point cloud were then proposed to improve flexibility of model deformation [21], but updating points needs enormous calculation. Recent years, hybrid models (models coupled surface meshes with internal point elements) were developed to improve incision rendering effect and real-time capability. Some scholars attempted constructing hybrid models to simulate cutting operation on cornea, liver, stomach, gallbladder and spleen [22–24]. Visual and haptic rendering effects were improved, but some problems remain: soft tissue organs were usually modeled as homogeneous material without considering the difference of biomechanical properties among micro-anatomic structures; In addition, since feedback force or friction force during cutting have not been studied too much, most of simulations cannot provide authentic tactile feedback [25]. Maxillofacial soft tissue is multilayer tissue consisting of skin, facia, muscle and mucosa. It possesses more complex characteristics compared with visceral organs. Schendel et al. constructed a facial soft tissue model instilled with skin stiffness parameters to simulate cleft lip repair [17]. Miki et al. presented a virtual surgical system for training submandibular glands excision. The submandibular gland and adjacent vessels were modeled and experimental force was incorporated for haptic rendering [18]. To our best knowledge, none of deformable force models of facial soft tissue for cutting simulation was proposed yet.

## Methods

**Fig. 1:**
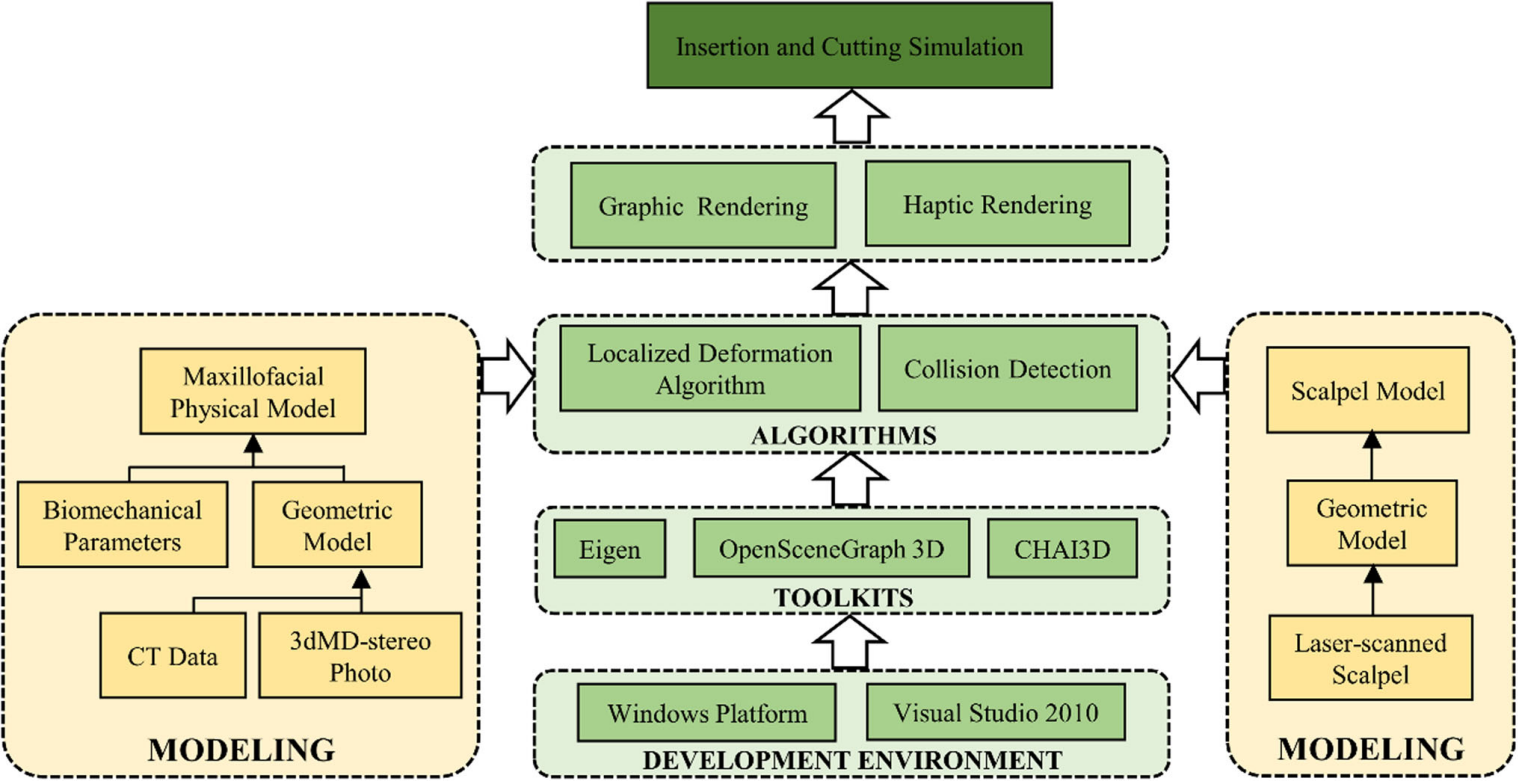
Architecture of the system

Visual Studio 2010 was chosen as the Integrated Development Environment (IDE). Some open-source toolkits based on C +  + were also involved. Eigen was used for matrix calculation. OpenSceneGraph and CHAI3D were used for 3D visualization and force feedback, respectively. Virtual maxillofacial physical model and scalpel model were constructed using raw data. Localized deformation and collision detection algorithm were designed to realize rapid calculation of deformation and feedback force. Omega 6 (Force Dimension, Swiss) was employed for haptic rendering. The architecture of the system is shown in Fig. 1. The following paragraphs state detailed methodology of each module.

### Data acquisition and physical model construction

A 25-year-old female with dento-maxillofacial deformity was selected as raw data source in this study.

#### Construction of geometric models

Craniofacial CT scans (0.625-mm-thick slices) of the patient were acquired (LightSpeed Ultra 16 spiral CT machine, GE Company, USA). Original data (DICOM format) were imported into Mimics software (Materialise Company, Belgium) and segmented as skeletal and soft tissue separately. Multiple layers (skin, fat, muscle and mucosa) of facial soft tissue were segmented as a whole, so that geometric model of facial soft tissue was single-layer structure. Segmentation was accomplished by manually adjusting Hounsfield threshold values to obtain the most accurate anatomic structures. 3D models of skeletal and soft tissue were reconstructed, respectively and exported as stereolithography (STL) files. The patient’s face was also captured using 3dMD face photogrammetric system (3dMD Inc., Atlanta, GA) and saved as MTL and OBJ format files. 3dMD stereo-photo was registered to CT data-reconstructed facial surface to include colored texture in the virtual model. Triangular meshes of facial surface were optimized for accurate deformation. Meshes of bone tissue were simplified to improve calculation speed. At last, the whole virtual maxillofacial model consisted of 149,409 triangle elements, 65,600 of which constructed facial surface. In addition, a scalpel with rounded No. 10 blade was scanned by 3D laser scanner and saved as STL format file for instrument modeling.

#### Incorporation of biomechanical parameters

**Fig. 2:**
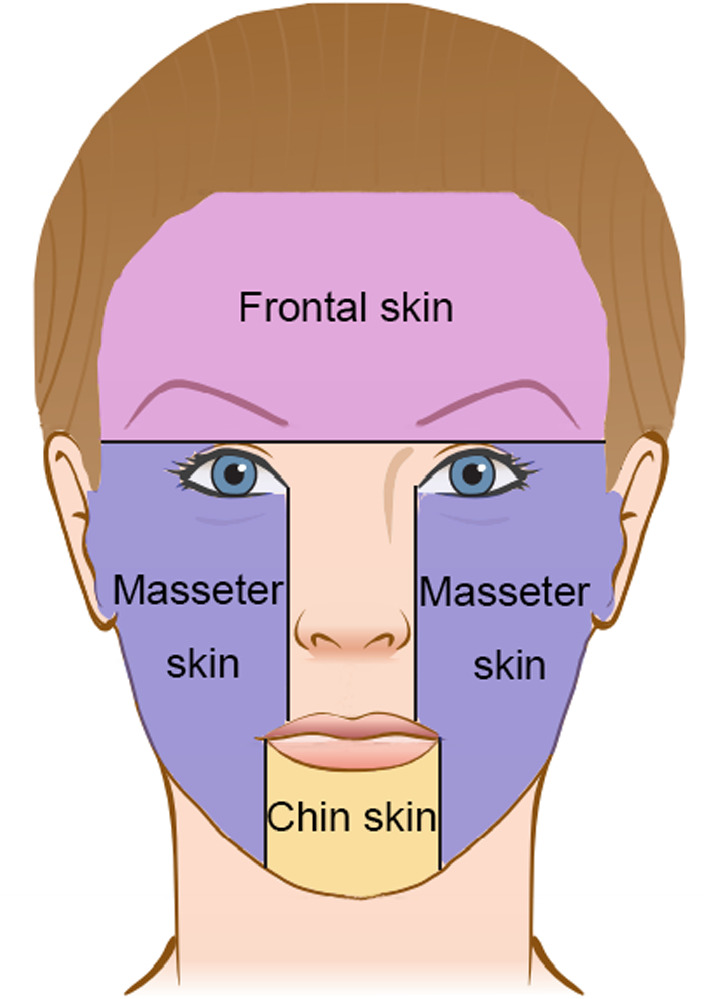
Anatomic distribution of facial soft tissue in virtual model. Facial skin was manually divided into frontal skin, masseter skin and chin skin. The division was simplified compared with actual anatomy

**Fig. 3:**
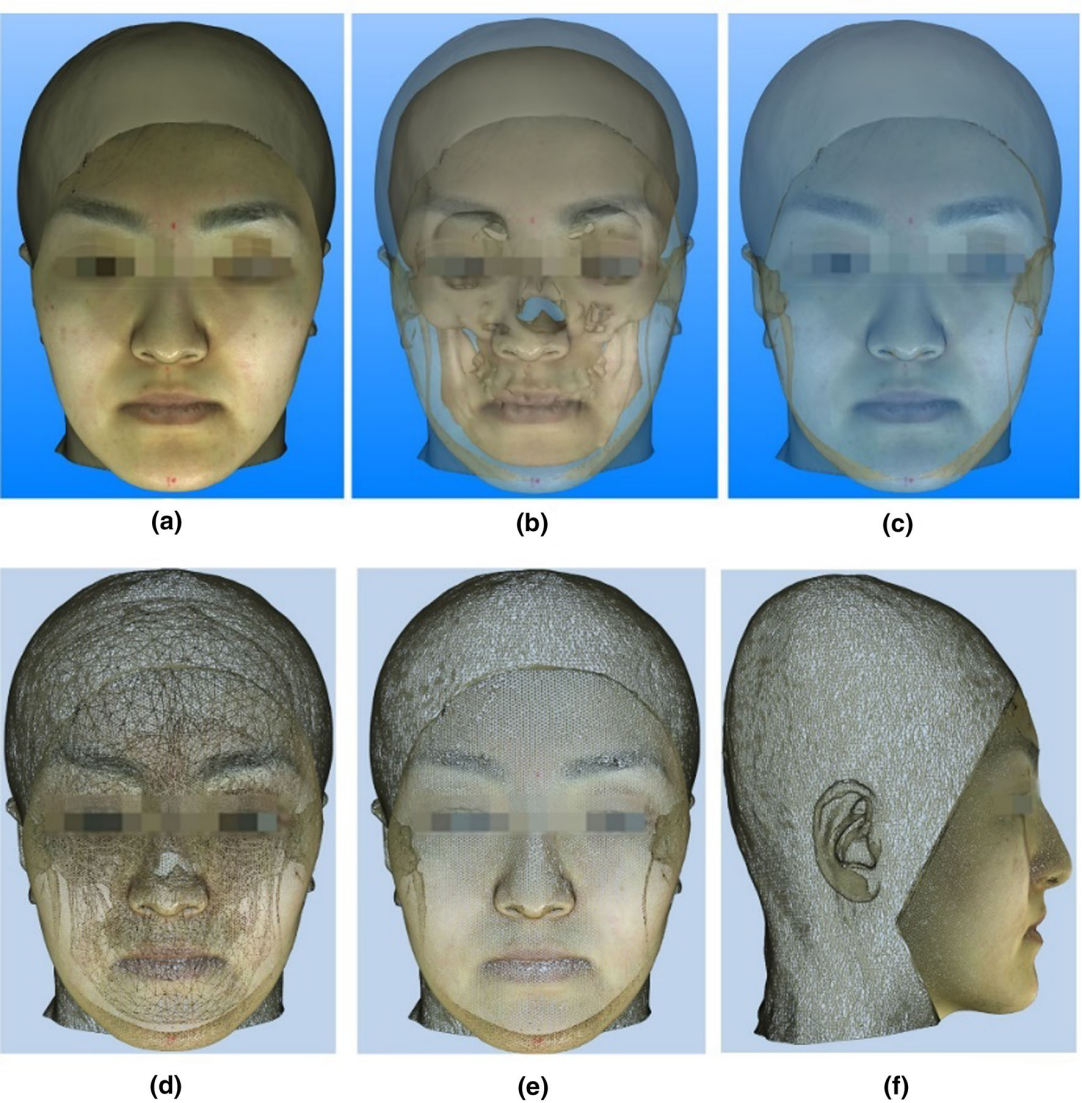
Maxillofacial physical model composed of skeletal and soft tissue. **a** Original display, **b** semitransparent display, **c** semitransparent display with invisible skeletal model, **d-f** triangular meshes of virtual model

The single-layer structure of facial soft tissue in geometric model would be incorporated with biomechanical properties of facial skin. According to previous study [19], facial skin exhibits different biomechanical properties in different anatomic regions, so boundary conditions were set in the virtual model based on coordinate values of vertexes in world coordinate system to simulate anatomic distribution of skin (Fig. 2). Insertion equations and cutting forces of each region were fitted correspondingly. Detailed parameters derived from experimental data of the female maxillofacial soft tissue in [19]. Then the construction of maxillofacial physical model was completed (Fig. 3).

### Localized deformation algorithm

To realize mechanical response of the virtual skin, stiffness matrix of spatial triangular mesh was constructed at first based on the method in [26]. Then adjacent coordinated deformation of spatial mesh was solved based on the analysis of geometric constraints between triangular elements. At the same time, the deformation range was restricted and localized by defining displacement boundary conditions to simplify the matrix. At last, shape, color and number of triangular elements were updated for visual rendering. The following are detailed procedures.

#### Construct stiffness matrix of spatial triangular mesh

Stiffness matrix of one triangular element can be defined as $K_{e}$,

1 $$ K_{e} = \lambda {\text{DKDK}} $$

where $\lambda$ is anti-deformation stiffness of the triangle element, $K$ is the global stiffness matrix and $D$ is a diagonal correction matrix, which is used to reflect the influence of surrounding elements on the element. Equation (1) is derived from [26]. $K_{e}$ is a 3 × 3 matrix. Triangle mesh $S$ or $S^{\prime}$ is composite of $n$ nodes, so that stiffness matrix of $S$ and $S^{\prime}$, namely $K_{S}$ and $\tilde{K}_{s}^{^{\prime}}$, is stacked $3n$-order matrix. The triangle mesh Laplace (TML) method proposed in [26] was used to construct and solve the stiffness matrix.

#### Solve adjacent coordinated deformation

Deformation of one specific triangular element is linked with directly exerted force and adjacent geometric constraints. Geometric constraints are connection nodes which build connectivity in two or more triangular elements. Adjacent triangular elements interacted when force is exerted or displacement occurred by the way of connection nodes. Therefore, connection nodes were used to calculate the transfer relationship of force and displacement among triangular elements. To solve adjacent coordinated deformation problems with connection relations, an initial displacement of the connection nodes is assumed. Then deformation of adjacent meshes can be solved, respectively. If equal forces in opposite direction are exerted at the connection node of two meshes, initial displacement is exactly correct deformation, and the calculation ends; otherwise, initial displacement value needs to be adjusted based on the resultant force at the connection node to obtain a new displacement vector. Iterative calculations would be performed for solution. The following are detailed derivation process.

Assuming there are a set of connection nodes in triangle mesh $S$ and $S^{\prime}$, namely $\left\{ V \right\} = \left\{ {V_{1} ,V_{2} , \ldots ,V_{n} } \right\}$ in $S$, $\{ V\}^{^{\prime}} = \left\{ {V_{1}^{^{\prime}} ,V_{2}^{^{\prime}} , \ldots ,V_{n}^{^{\prime}} } \right\}$ in $S^{\prime}$. The initial displacement of each node was set as the midpoint of corresponding connection node in $S$ and $S^{\prime}$. Hence, the initial displacement of $\left\{ {V_{1} ,V_{2} , \ldots ,V_{n} } \right\}$ is:

2 $$ \{ \delta \}_{0} = \left\{ {\delta_{01} ,\delta_{02} ,...,\delta_{0n} } \right\} = \left\{ {\frac{{V_{1}^{^{\prime}} - V_{1} }}{2},\frac{{V_{2}^{^{\prime}} - V_{2} }}{2},...,\frac{{V_{n}^{^{\prime}} - V_{n} }}{2}} \right\} $$

The initial displacement of $\left\{ {V_{1}^{^{\prime}} ,V_{2}^{^{\prime}} , \ldots ,V_{n}^{^{\prime}} } \right\}$ is:

3 $$ \{ \delta \}_{0}^{^{\prime}} = \left\{ { \delta_{01}^{^{\prime}} ,\delta_{02}^{^{\prime}} ,...,\delta_{0n}^{^{\prime}} } \right\} = \left\{ {\frac{{V_{1} - V_{1}^{^{\prime}} }}{2},\frac{{V_{2} - V_{2}^{^{\prime}} }}{2},...,\frac{{V_{n} - V_{n}^{^{\prime}} }}{2}} \right\} $$

Use the values of $\{ \delta \}_{i}$ and $\{ \delta \}_{i}^{^{\prime}}$ to perform iterative calculations to determine whether the value of the force at the connection node meets:

4 $$ \frac{{\sqrt {\left| {F_{i1} + F_{i1}^{^{\prime}} } \right|^{2} + \left| {F_{i2} + F_{i2}^{^{\prime}} } \right|^{2} + ... + \left| {F_{{{\text{in}}}} + F_{{{\text{in}}}}^{^{\prime}} } \right|^{2} } }}{n} \le \varepsilon $$

where $\varepsilon$ is the convergence error. If this condition is met, the current displacement values ${ }\{ \delta \}_{i}$ and $\{ \delta \}_{i}^{^{\prime}}$ are exactly the actual displacement values of the node. Otherwise, the iterative displacement values of the connection nodes in $S$ and $S^{\prime}$ are calculated using the equation:

5 $$ \left\{ {\begin{array}{*{20}c} {\{ \delta \}_{i + 1} = \{ \delta \}_{i} + 2(\tilde{K}_{s} + \tilde{K}_{s}^{^{\prime}} )^{ - 1} (\{ F\}_{i}^{^{\prime}} - \{ F\}_{i} )} \\ {\{ \delta \}_{i + 1}^{^{\prime}} = \{ \delta \}_{i}^{^{\prime}} + 2(\tilde{K}_{s} + \tilde{K}_{s}^{^{\prime}} )^{ - 1} (\{ F\}_{i}^{^{\prime}} - \{ F\}_{i} )} \\ \end{array} } \right. $$

where $\{ F\}_{i + 1}$ and $\{ F\}_{i + 1}^{^{\prime}}$ are calculated in iterative process until the error judgment condition is met.

#### Define boundary conditions to localize deformation range

Since facial soft tissue is elastic material, it has complicated deformation boundaries. Two types of displacement boundary conditions were used when solving the matrix: (1) the displacement value of the collision point between the scalpel and soft tissue. This value can be obtained from the state of previous frame; and (2) deformation range was localized. During insertion or cutting process, the nodes far away from the blade–tissue collision point were affected slightly, so they were neglected and the deformation was localized in a defined range to simplify the calculation. The definition of deformation range is as follows.

**Fig. 4:**
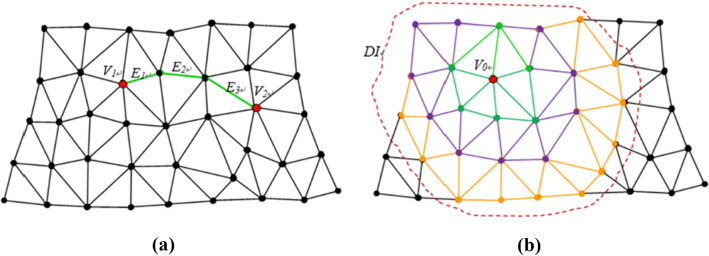
**a** “Side distance” of spatial triangular meshes; **b** *DI* of node *V0*

The minimum distance between two nodes of one spatial triangular mesh can be expressed as:

6 $$ l_{{{\text{min}}}} = \min \left( {l_{V1,V2} } \right) = \min \left( {\sum E_{i} } \right) = \mathop \sum \limits_{i = 1}^{\min N} E_{i} $$

where $$ l_{V1,V2}$$ is “side distance,” namely the sum of length of all sides needed for node *V*1 access to *V*2; $$E_{i}$$ is one of the sides in the way of node *V*1 to *V*2 (*i* ≥ 1); and $$\min N$$ is the minimum number of sides from *V*1 to *V*2 (Fig. 4a).

Assuming one node in aforementioned mesh is exerted with force or occurred displacement, neighboring nodes would be affected and occurred displacement. The area consisted of these displaced nodes is defined as ${\text{DI}}$, short for *influenced district.* The range of ${\text{DI}}$ can be represented by $\min N$. For example, ${\text{DI}}$ of node *V0* is 3 in Fig. 4b. According to the experimental results in [19], ${\text{DI}}$ enlarged as force, displacement or elastic modulus increased. It can be expressed as:

7 $$ {\text{DI}} = f_{{{\text{DI}}}} \left( {\varepsilon ,E_{m} ,d} \right) $$

where $\varepsilon$ is displacement value which represented the influence of exerted force; $E_{m}$ is averaged elastic modulus of epidermis and dermis; and $d$ is thickness of the skin. Displacement value can be converted from millimeter to "side distance." Therefore, when solving basic equations, the displacement of nodes beyond ${\text{DI}}$ can be directly set as zero, and calculation costs was significantly decreased. In actual implementation process, the number of elements involved in the calculation was usually 200–500, which promised a converged results. Besides, in the algorithm, each iteration uses the state of the previous frame as the initial value which means that the displacement value in each iteration is small and therefore promises the convergence to some extent. In order to ensure the system robustness, the maximum number of iterations was set to 500, exceeding which the iteration was terminated. Eigen conjugate gradient was employed for solving the matrix.

#### Visual rendering

To visually render an incision for three-dimensional display, once the system detected blade–tissue collision nodes in frame loop, the direction of the scalpel movement will be tracked and the displacement of the nodes in $DI$ will be calculated and updated. When current exerted force meets puncture conditions, the constraints of the nodes will be unleashed. Then distorted meshes were updated, and new meshes were generated according to calculated displacement of nodes. At the same time, color and texture of triangular meshes in the incision area were changed in visual module, making the incision more vivid. OpenSceneGraph toolkit was used for 3D visual rendering.

### Collision detection algorithm

As virtual scalpel was modeled with triangular surface meshes as well, collision detection between scalpel and soft tissue, scalpel and bone tissue was performed on triangular mesh models. The system employed axis aligned bounding box (AABB) method for collision detection. The collision model was organized as binary tree structure. Interference between bounding boxes and that between bounding boxes and triangular meshes were, respectively, completed by nominal radius theorem (NRT) and separating axis theorem (SAT). The purposes of collision detection in this system include: firstly, detecting collision point to determine penetration depth of the blade which is required as input for rapid calculation of soft tissue deformation and feedback force, and secondly detecting bone–blade collision to intercept continually blade’s penetration.

### Haptic feedback

Force Dimension’s omega 6 was used for haptic feedback. When operator manipulated virtual scalpel through the handle of omega 6, force response will be triggered once blade–tissue collision was detected. Calculated force was outputted to operator immediately. Considering maximum feedback force of omega 6 is nearly 12 N, and original modeling feedback force was compromised by multiplying a coefficient. CHAI3D toolkit (Computer Haptics and Active Interfaces, Force Dimension, Swiss) was used for interactive real-time haptic simulation.

## Results

**Fig. 5:**
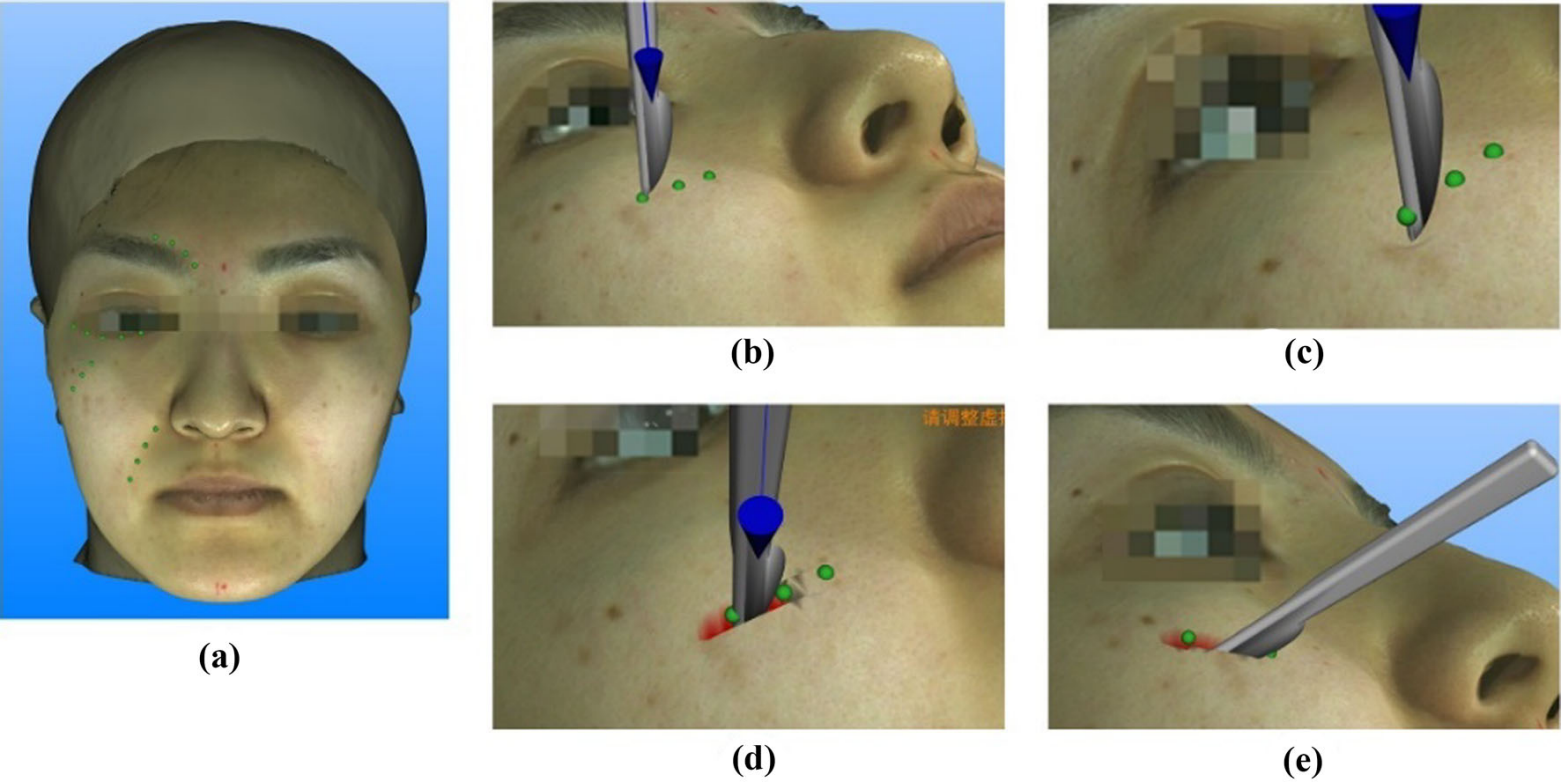
Three steps of making an incision in virtual surgery. **a** Planning an incision path; **b** before insertion, the scalpel was perpendicular to facial surface; **c** insertion; **d** insertion completed; **e** cutting process, the scalpel was manually adjusted to 45° oblique to facial surface

Making an incision in virtual surgery was composed of three steps based on actual operation procedure: planning an incision path, insertion and cutting (Fig. 5). Operators can digitize landmarks to define the path and length of an incision at first. Click “next step” and a scalpel with rounded No.10 blades showed up above the first landmark, perpendicular to facial surface. Then, the operator could grasp the handle of omega 6 and puncture facial soft tissue. During insertion process, the system recorded the penetration depth of the blade in frame loop as input to calculate real-time exerted force and displacement of triangular meshes. As force gradually enhanced and the tip of blade moving down, the skin occurred elastic deformation and gradually sank (Fig. 5c). If the scalpel left the skin surface with no contact at this moment, triangular meshes would restore to original shape. When the pressure on the collision point was larger than maximum stiffness, meshes ruptured and cannot restore (Fig. 5d). Feedback force dropped to zero at this time and the operator had “breakthrough” feeling. Next, the operator manipulated the scalpel to 45° oblique position to initiate cutting process. Thereafter, the operator can move the scalpel manually along with the predefined path and perform incision (Fig. 5e). When reached the end of the incision, operator can lift up the scalpel to terminate the cutting process. Aforementioned procedures can be performed using mouse or the handle of omega 6. The average 3D scene update rate of the system was 59.3 FPS (frames per second). The simulation calculation update rate is slightly slower (about 20 Hz-50 Hz). Haptic update rate is around 500–1000 Hz. The response time of the system was 0.05–0.1 s. Overall, visual and haptic refresh rate in this system guaranteed real-time interaction between operators and the system.

**Fig. 6:**
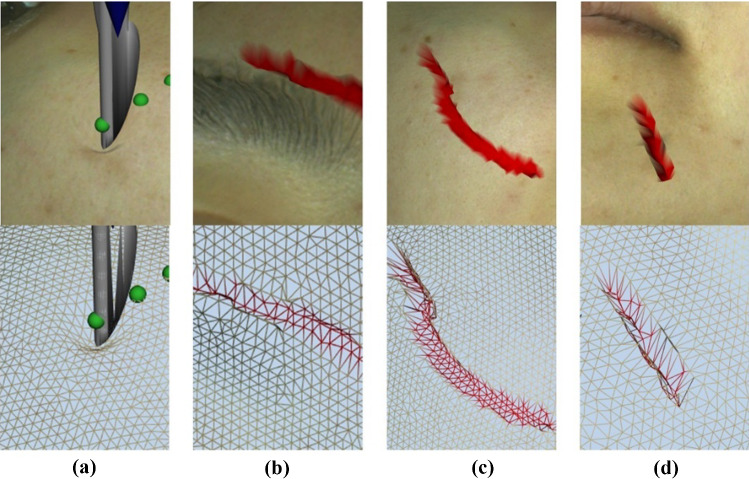
Deformation of insertion and cutting in different regions of facial soft tissue. **a** Insertion; **b** cutting on frontal skin; **c** cutting on masseter skin; **d** cutting on chin skin

**Fig. 7:**
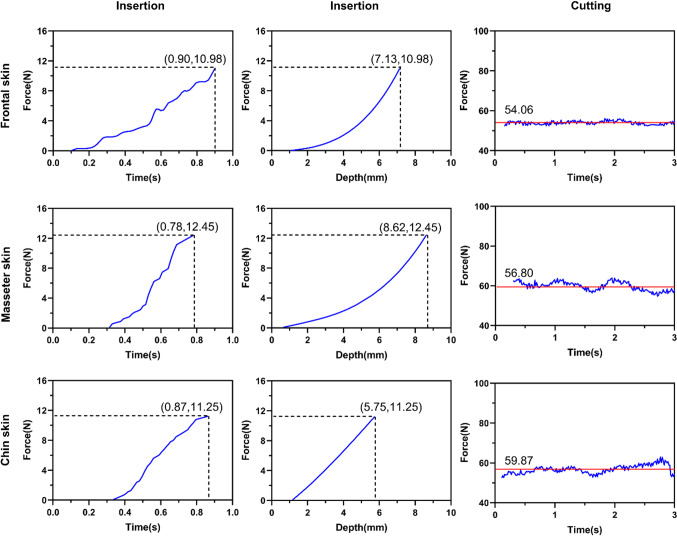
Insertion time–force curves and depth–force curves and cutting forces of facial soft tissue in different regions. Red straight lines showed the means of feedback force when performed cutting

Insertion and cutting in different regions of facial soft tissue were simulated using omega 6 (Fig. 6). Calculated feedback force of the maxillofacial physical model was recorded for quantitative validation. Force–time curves and force–depth curves of insertion, and force–time curves of cutting were plotted (Fig. 7). Insertion process usually lasted less than one second. Before skin was punctured, maximum feedback force values were, respectively, 10.98, 12.45 and 11.25 N for frontal, masseter and chin regions. When the tissue was punctured, the depths of blade were 7.13, 8.62 and 5.75 mm in frontal, masseter and chin regions, respectively. Force–time curves of cutting were plotted using 3-s-long data extracted from whole cutting process. The means of cutting force of frontal, masseter and chin skin were 54.06, 56.80 and 59.87 N, respectively.

**Fig. 8:**
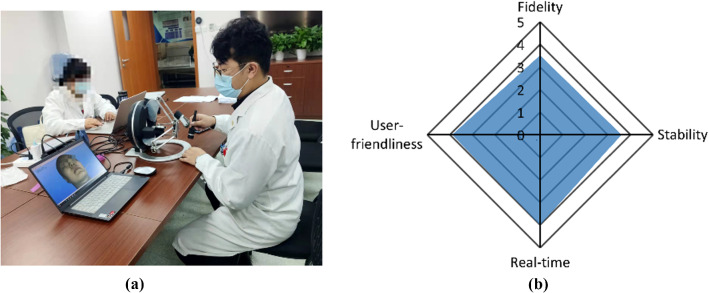
Qualitative evaluation about system performance. **a** Virtual surgical simulation; **b** evaluation results

Ten experienced surgeons in the Department of Oral and Craniomaxillofacial Surgery were recruited for qualitative evaluation (Fig. 8a). After usage demonstration, this system was used to simulate incisions on facial soft tissue. Then, a 1–5 Likert scale questionnaire which consisted of ten questions about four key features of a virtual surgery system (*Supplementary data 1*) was designed for qualitative evaluation. The mean grades were 3.53 for fidelity of visual and haptic feedback, 3.60 for stability, 4.00 for real-time capability and 3.90 for user-friendliness (Fig. 8b). Detailed grade distribution of each question in the questionnaires can be obtained in *Supplementary data 2.*

## Discussion

Making incisions in facial soft tissue is a delicate procedure during surgery. It involves perception of feedback force and depth of the blade. It also requires firm, continuous manipulation of scalpel. In this study, we developed a haptics-enabled virtual surgical system based on a maxillofacial physical model, to simulate incisions in facial soft tissue. Moreover, the computed feedback force of the physical model during simulation was evaluated and good fidelity of force feedback was obtained.

The design of the virtual surgical system considered special characteristics of maxillofacial soft tissue. Firstly, the system enabled operators to plan the path and length of incisions through digitizing landmarks on face. Planning incisions previously is necessary for most of maxillofacial surgeries which involve flap design such as cheiloplasty. In addition, incisions on face are usually curved to keep consistent with Langer’s lines of facial skin [27]. Preset path helps surgeons especially novice surgeons make incisions precisely. The maxillofacial model in this system is actually a universal model allowing random access points and arbitrary cutting paths. The operator was given the option of skipping landmark digitization step and manually placing the scalpel at their intended location. Secondly, virtual scalpel position will affect the operation. When inserting, the handle of scalpel is perpendicular to facial surface; after insertion, the scalpel needs to be manually adjusted to 45° oblique to facial surface to initiate cutting process (Fig. 5). In actual surgery, scalpel of 90°-position helps operator orient the beginning of incision accurately and penetrate keratinized epidermis. Scalpel of 45°-position ensures the operator’s flexible and fluent motion to maintain constant cutting depth. Moreover, the blade should be kept perpendicular to facial surface during cutting to obtain squared incision edges, which can prevent tissue necrosis of the wound edge and benefit healing [1]. In the system, if the operator digitized landmarks, the scalpel will be automatically adjusted to the correct position. If the operator skips this step, scalpel needs to be manually adjusted for the whole process. During simulation in the latter manner, when the scalpel was not manually adjusted to perpendicular position, simulated incisions presented as wider and more jagged shape just like more damaged tissue in actual operation. Thirdly, bone–scalpel collision can be detected in simulation to indicate the blade reached bone surface and stop it moving deeper. When the collision occurred, the haptic device gave the operator "hard and resistant" feeling. Fourthly, facial soft tissue can be set as semitransparent display mode so that operators can intuitionally observe the depth of blade and feel feedback force at the same time. Aforementioned characteristics were not reported in previous study. These adaptive designs enabled our system more suitable for incisions simulation, especially on facial skin.

To validate the accuracy of calculated feedback force of the maxillofacial physical model proposed in this study, incisions on frontal, masseter and chin skin were simulated with haptic device by operators (Fig. 6). Computed feedback forces were recorded in system simultaneously (Fig. 7). Since different operators manipulated the scalpel with different speed (obviously we cannot control this variable) and chose insertion sites as they want (different sites mean different skin thickness, even different biomechanical parameters), data plotted in Fig. 7 were randomly chosen from all records as exemplification of biomechanical properties in different facial region. Force data acquired in corresponding experiments on fresh cadavers in [19] were viewed as benchmark of the model validation. The force–depth curves of insertion were consistent with polynomial equations acquired in previous study [19]. The curves, differed from different regions, exhibited the difference of biomechanical properties. Acquired maximum stiffness values of facial skin in simulation tests were larger than experimental results in previous study [19]. The reason might be that segmented soft tissue structures were completely simulated as skin in the maxillofacial model to simplify the calculation. As a result, the thickness of simulated skin in each region was bigger than actual skin. Since insertion depth increased, the maximum stiffness was accordingly increased. Based on the premise that the depth of blade does not change, cutting force is constant. However, the speed of blade’s moving also affects force values. Due to the change of cutting speed, feedback force fluctuated around a specific value (Fig. 7). In the previous study [19], cutting force was measured by piezoelectric dynamometer (Kistler 9256C2, Switzerland) and the results showed cutting force was composed of vertical force in Z axis and horizontal force in Y axis. In virtual surgery, feedback cutting forces were resultant forces of Y and Z axis. At the same time, feedback force the operator perceived in simulation was reduced by multiplying a coefficient, for the reason that actual cutting force ranges from 40 to 80 N while the maximal feedback force of omega 6 is about 12 N. In general, through comparing the experimental data in this virtual system with acquired force data in [19], good fidelity of feedback force was achieved based on this virtual model.

Developing virtual surgical simulation system always faces challenges in balancing accuracy and real-time capability. Accurate deformation and haptic feedback require physical modeling of bio-tissue, which means integration of real mechanical parameters and geometric models so that tissue’s motion or deformation can conform to physical rules [28]. For elastic deformation of soft tissue, classic and mature methods like mass–spring method (MSM) or finite element method (FEM) can guarantee high accuracy, but time consumed on precalculation is too long to meet real-time demand of rapid deformation [29]. The aim of this research was to propose a rapid feedback force calculation model that can be applied in virtual surgery training. Though the calculation accuracy was compromised a little (especially in terms of deformation accuracy) to meet real-time requirements, calculation results showed that the direction, magnitude and trend of force and deformation were consistent with previous experimental data. Hence, operators can still acquire realistic experience of making incisions on facial skin through this system. Tactile feedback was proved to be meaningful for operators to practice skills and improve their performance [13, 30]. In most of proposed virtual surgical systems, haptic feedback was not realized or haptic rendering was not authentic for the reasons that interaction between surgical instruments and bio-tissue has not been studied too much and real deformation and experimental force data are insufficient. Based on experimental data measured on fresh cadavers, haptic feedback in our proposed system showed good fidelity and provided operators immersive and realistic training experience.

## Conclusion and further perspective

In this study, a haptics-enabled virtual surgical system was developed to simulate surgical incisions of facial soft tissue and the prototype was evaluated quantitatively and qualitatively. Composite maxillofacial physical model integrated with real force data promised the fidelity of haptic feedback. Localized deformation and AABB-based collision detection algorithm guaranteed the computation efficiency of simulation. The developed virtual surgical system provided users real-time and realistic interaction, and demonstrated excellent performance in medical training.

More researches on specimen and actual surgery need to be done to analyze the deformation behavior of soft tissue. In follow-up research work, we will optimize the model in terms of calculation accuracy and design more efficient algorithms for soft tissue surgery simulation. Visual effects of incisions can be sharper and smoother. More layers of soft tissue such as muscles and mucosa need to be simulated to enhance realistic tactile sensation and expand system’s function. Operation on maxillofacial bones can be integrated into the system as well. In addition, as a virtual surgical training system, a performance score module should be developed to evaluate operators’ performance automatically and objectively.

## Supplementary Information

Below is the link to the electronic supplementary material.

Supplementary file1 (DOCX 21 kb)

Supplementary file2 (DOCX 26 kb)

## References

[1] Hupp JR, Hupp JR, Tucker MR, Ellis E (2019). Principles of surgery. Contemporary oral and maxillofacial surgery.

[2] Pohlenz P, Grobe A, Petersik A, Von Sternberg N, Pflesser B, Pommert A, Hohne K-H, Tiede U, Springer I, Heiland M (2010). Virtual dental surgery as a new educational tool in dental school. J Cranio-Maxillofac Surg.

[3] Zhang B, Li S, Gao S, Hou M, Chen H, He L, Li Y, Guo Y, Wang E, Cao R, Cheng J, Li R, Zhang K (2020). Virtual versus jaw simulation in Oral implant education: a randomized controlled trial. BMC Med Educ.

[4] Chen X, Hu J (2018). A review of haptic simulator for oral and maxillofacial surgery based on virtual reality. Expert Rev Med Devices.

[5] Gosselin F, Bouchigny S, Megard C, Taha F, Delcampe P, d'Hauthuille C (2013). Haptic systems for training sensorimotor skills: a use case in surgery. Robot Auton Syst.

[6] Wu FL, Chen XJ, Lin YP, Wang CT, Wang XD, Shen GF, Qin J, Heng PA (2014). A virtual training system for maxillofacial surgery using advanced haptic feedback and immersive workbench. Int J Med Robot Comput Assist Surg.

[7] Arikatla, V S, Tyagi, M, Enquobahrie, A, Tung, N, Blakey, G H, White, R, and Paniagua, B (2018) High Fidelity Virtual Reality Orthognathic Surgery Simulator*.* Medical Imaging 2018: Image-Guided Procedures, Robotic Interventions, and Modeling 10576: UNSP 1057612 10.1117/12.2293690 10.1117/12.2293690 PMC6028926 29977103

[8] Lin YP, Yu DD, Chen XJ, Wang XD, Shen GF, Wang CT (2014). Simulation and evaluation of a bone sawing procedure for orthognathic surgery based on an experimental force model. J Biomech Eng-Trans Asme.

[9] Sohmura T, Hojo H, Nakajima M, Wakabayashi K, Nagao M, Iida S, Kitagawa T, Kogo M, Kojima T, Matsumura K, Nakamura T, Takahashi J (2004). Prototype of simulation of orthognathic surgery using a virtual reality haptic device. Int J Oral Maxillofac Surg.

[10] Yu HB, Cheng J, Cheng AHA, Shen SG (2011). Preliminary study of virtual orthognathic surgical simulation and training. J Craniofac Surg.

[11] Zaragoza-Siqueiros J, Medellin-Castillo HI, de la Garza-Camargo H, Lim T, Ritchie JM (2019). An integrated haptic-enabled virtual reality system for orthognathic surgery planning. Comput Methods Biomech Biomed Eng.

[12] Wang Q, Chen H, Wu W, Jin H-Y, Heng P-A (2012). Real-time mandibular angle reduction surgical simulation with haptic rendering. IEEE Trans Inf Technol Biomed.

[13] Yan Y, Li Q, Wang Q, Peng Y (2018). Real-time bone sawing interaction in orthopedic surgical simulation based on the volumetric object. J Vis.

[14] Chen XJ, Sun PJ, Liao DH (2018). A patient-specific haptic drilling simulator based on virtual reality for dental implant surgery. Int J Comput Assist Radiol Surg.

[15] Olsson P, Nysjo F, Hirsch JM, Carlbom IB (2013). A haptics-assisted cranio-maxillofacial surgery planning system for restoring skeletal anatomy in complex trauma cases. Int J Comput Assist Radiol Surg.

[16] Olsson P, Nysjo F, Rodrigez-Lorenzo A, Thor A, Hirsch JM, Carlbom IB (2015). Haptics-assisted virtual planning of bone, soft tissue, and vessels in fibula osteocutaneous free flaps. Plast Reconstr Surg-Glob Open.

[17] Schendel S, Montgomery K, Sorokin A, Lionetti G (2005). A surgical simulator for planning and performing repair of cleft lips. J Cranio-maxillofac Surg.

[18] Miki T, Iwai T, Kotani K, Dang J, Sawada H, Miyake M (2016). Development of a virtual reality training system for endoscope-assisted submandibular gland removal. J Cranio-Maxillofac Surg.

[19] Zhuang Y, Chen J, Liu QC, Zou F, Lin YH, An QL, Yu HB (2021). Preliminary study on mechanical characteristics of maxillofacial soft and hard tissues for virtual surgery. Int J Comput Assist Radiol Surg.

[20] Pietroni N, Ganovelli F, Cignoni P, Scopigno R (2009). Splitting cubes: a fast and robust technique for virtual cutting. Vis Comput.

[21] Jin X, Joldes GR, Miller K, Yang KH, Wittek A (2014). Meshless algorithm for soft tissue cutting in surgical simulation. Comput Methods Biomech Biomed Eng.

[22] Pan J, Yan S, Qin H, Hao A (2016). Real-time dissection of organs via hybrid coupling of geometric metaballs and physics-centric mesh-free method. Vis Comput.

[23] Cheng Q, Liu PX, Lai P, Xu S, Zou Y (2018). A novel haptic interactive approach to simulation of surgery cutting based on mesh and meshless models. J Healthcare Eng.

[24] Zou Y, Liu PX, Wu D, Yang X, Xu S (2019). Point primitives based virtual surgery system. IEEE Access.

[25] Lindblad, A and Turkiyyah, G (2007) A physically-based framework for real-time haptic cutting and interaction with 3D continuum models*.* Proceedings of the 2007 ACM symposium on Solid and physical modeling 421–429. 10.1145/1236246.1236307

[26] Cheng HC, Wu DL, Fan XM (2019). Modeling and simulation of sheet-metal part deformation in virtual assembly. J Ambient Intell Humaniz Comput.

[27] Paul SP (2018). Biodynamic excisional skin tension lines for surgical excisions: untangling the science. Ann R Coll Surg Engl.

[28] Maliha SG, Diaz-Siso JR, Plana NM, Torroni A, Flores RL (2018). Haptic, physical, and web-based simulators: are they underused in maxillofacial surgery training?. J Oral Maxillofac Surg.

[29] Zhang XR, Duanl JL, Zhu LF, Kavan L (2018). A virtual puncture surgery system based on multi-layer soft tissue and force mesh. CMC-Comput Mater Contin.

[30] Medellin-Castillo HI, Zaragoza-Siqueiros J, Govea-Valladares EH, de la Garza-camargo H, Lim T, Ritchie JM (2020). Haptic-enabled virtual training in orthognathic surgery. Virtual Real.

