# Supplementary material for: Development of a maxillofacial virtual surgical system based on biomechanical parameters of facial soft tissue
Source: Int J Comput Assist Radiol Surg. 2022 May 15;17(7):1201–11. doi: 10.1007/s11548-022-02657-5 (PMC9206636; doi:10.1007/s11548-022-02657-5)
Supplement: Supplementary file 1 — Supplementary file1 (DOCX 21 kb) [file 11548_2022_2657_MOESM1_ESM.docx]

***International Journal of Computer Assisted Radiology and Surgery***

**Development of** **a maxillofacial virtual surgical system based on biomechanical parameters of facial soft tissue**

Mengjia Cheng DDS^a, b, c,1^, Yu Zhuang DDS^a, b, c,1^, Hanjiang Zhao DDS^a, b, c^, Meng Li^a, b, c^, Lingfeng Fan^b,c,d.*^, Hongbo Yu DDS. MD ^a, b, c.*^

a. Department of Oral and Cranio-maxillofacial Surgery, Shanghai Ninth People’s Hospital, College of Stomatology, Shanghai Jiao Tong University School of Medicine, Shanghai 200011, China;

b. National Clinical Research Center for Oral Diseases, Shanghai 200011, China;

c. Shanghai Key Laboratory of Stomatology & Shanghai Research Institute of Stomatology, Shanghai 200011, China.

d. Department of Radiology, Shanghai Ninth People’s Hospital, College of Stomatology, Shanghai Jiao Tong University School of Medicine, Shanghai 200011, China

^1^ Mengjia Cheng and Yu Zhuang have contributed equally on this work.

^*^ Corresponding author: Email address: yhb3508@163.com (Yu H), 847472535@qq.com (Fan L).

**面部软组织手术仿真系统性能评估问卷**

测试者您好，请根据个人使用情况完成本问卷。每个问题后面请在最符合的一项处打勾，感谢您的测试和反馈！

| **问题** | **非常**  **不同意** | **不同意** | **不确定** | **同 意** | **非常**  **同意** |
| --- | --- | --- | --- | --- | --- |
| 1.面部解剖结构清楚，色泽及纹理逼真 |  |  |  |  |  |
| 2.穿刺时软组织产生的形变真实自然 |  |  |  |  |  |
| 3.手术切口视觉渲染真实自然 |  |  |  |  |  |
| 4.穿刺及切割中能感受到明显的力的变化，符合真实手术场景 |  |  |  |  |  |
| 5.力反馈设备的手柄可以自由控制手术刀 |  |  |  |  |  |
| 6.系统界面简洁易懂，上手快 |  |  |  |  |  |
| 7.力反馈设备手柄移动自由流畅，使用过程中没有感受到抖动或卡顿。 |  |  |  |  |  |
| 8.操作过程中软组织的形变过程流畅，没有卡顿 |  |  |  |  |  |
| 9.刺破皮肤前，随着我用手术刀施加力变大或变小，皮肤的形变也增加或减少，我感受到的反馈力同时也有相应的变化 |  |  |  |  |  |
| 10.切割皮肤时，我可以随心控制切口轨迹和长度，控制切割动作的开始与停止 |  |  |  |  |  |

**Performance Evaluation Questionnaire of a maxillofacial virtual surgical system**

Dear volunteers, please complete this questionnaire based on your own experience. After each question, please tick the most suitable option in the table. Thank you for your testing and feedback!

| **Questions** | **Strongly disagree** | **Disagree** | **Neither/Nor agree** | **Agree** | **Strongly agree** |
| --- | --- | --- | --- | --- | --- |
| 1. Anatomical structures are clear, with vivid color and texture. |  |  |  |  |  |
| 2.The visual rendering of soft tissue deformation during insertion is real and natural. |  |  |  |  |  |
| 3.The visual rendering of soft tissue incisions is real and natural. |  |  |  |  |  |
| 4.Obvious changes in force can be perceived during insertion and cutting, and feedback force accords with real surgical situations. |  |  |  |  |  |
| 5.the handle of haptic device can freely manipulate the virtual scalpel. |  |  |  |  |  |
| 6. The system interface is simple and easy to understand, quick to get started. |  |  |  |  |  |
| 7. The handle of the haptic device moved freely and smoothly, and there was no jitter or freeze during use. |  |  |  |  |  |
| 8. During the virtual operation, the deformation of the soft tissue is smooth and there is no freeze frame. |  |  |  |  |  |
| 9. Before the skin was pierced, as the force I exerted with the scalpel increased or decreased, the deformation of the skin increased or decreased, and the feedback force I felt also changed accordingly. |  |  |  |  |  |
| 10. When cutting the skin, I can control the trajectory and length of the incision freely, and initialize or end the cutting action freely. |  |  |  |  |  |

Note: Each question had five answers for participants to choose: strongly disagree (Grade marked as 1), disagree (Grade=2), Neither/Nor agree (Grade=3), agree (Grade=4), strongly agree (Grade=5).
