# Supplementary material for: Development of a maxillofacial virtual surgical system based on biomechanical parameters of facial soft tissue
Source: Int J Comput Assist Radiol Surg. 2022 May 15;17(7):1201–11. doi: 10.1007/s11548-022-02657-5 (PMC9206636; doi:10.1007/s11548-022-02657-5)
Supplement: Supplementary file 2 — Supplementary file2 (DOCX 26 kb) [file 11548_2022_2657_MOESM2_ESM.docx]

***International Journal of Computer Assisted Radiology and Surgery***

**Development of** **a maxillofacial virtual surgical system based on biomechanical parameters of facial soft tissue**

Mengjia Cheng DDS^a, b, c,1^, Yu Zhuang DDS^a, b, c,1^, Hanjiang Zhao DDS^a, b, c^, Meng Li^a, b, c^, Lingfeng Fan^b,c,d.*^, Hongbo Yu DDS. MD ^a, b, c.*^

a. Department of Oral and Cranio-maxillofacial Surgery, Shanghai Ninth People’s Hospital, College of Stomatology, Shanghai Jiao Tong University School of Medicine, Shanghai 200011, China;

b. National Clinical Research Center for Oral Diseases, Shanghai 200011, China;

c. Shanghai Key Laboratory of Stomatology & Shanghai Research Institute of Stomatology, Shanghai 200011, China.

d. Department of Radiology, Shanghai Ninth People’s Hospital, College of Stomatology, Shanghai Jiao Tong University School of Medicine, Shanghai 200011, China

^1^ Mengjia Cheng and Yu Zhuang have contributed equally on this work.


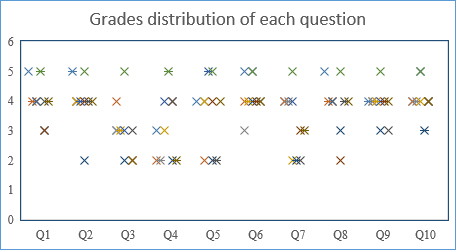
^*^ Corresponding author: Email address: yhb3508@163.com (Yu H), 847472535@qq.com (Fan L).

**Supplementary Data 2**. Grades distribution of each question in the questionnaires. "×" (namely grades) with same color means they were marked by the same participant.

Note: Question 1-4 were for fidelity of visual and haptic feedback, Q5-6 for user-friendliness, Q7-8 for stability, Q9-10 for real-time capability. Each question had five answers for participants to choose: strongly disagree (Grade was marked as 1), disagree (Grade=2), Neither/Nor agree (Grade=3), agree (Grade=4), strongly agree (Grade=5).
